# Back to basics with newer technology: Should we focus on reducing work of breathing earlier?

**DOI:** 10.3389/fmed.2022.1070517

**Published:** 2022-12-02

**Authors:** Christopher Sciarretta, Jeremy Greenberg, Kara D. Wyatt, Jessica S. Whittle

**Affiliations:** ^1^Department of Emergency Medicine, University of Tennessee College of Medicine, Chattanooga, TN, United States; ^2^Department of Respiratory, Critical Care, and Sleep Medicine, University of Tennessee, Chattanooga/Erlanger Health, Chattanooga, TN, United States; ^3^Scientific Consultant, Chattanooga, TN, United States; ^4^Department of Emergency Medicine, University of Tennessee - Chattanooga, Chattanooga, TN, United States; ^5^Vapotherm, Inc., Exeter, NH, United States

**Keywords:** sepsis, high-velocity therapy, critical care, high flow oxygen, high flow nasal cannula, acute respiratory failure, high-velocity nasal insufflation

## Abstract

The typical approach to management of respiratory distress is focused on oxygen supplementation. However, additional oxygen alone does not improve outcomes, particularly in critically ill patients. Instead, supplemental oxygen can be associated with increased morbidities. We present the hypothesis that clinicians should focus on reducing the work of breathing early in the course of critical illness. Rather than simply supplementing oxygen, newer technologies including high flow nasal oxygen, may be utilized to increase the efficiency of gas exchange. By reducing the work of breathing, the cardiac workload can be reduced, thus relieving some excess physiologic stress and supporting the critically ill patient. To illustrate this point, we provided three clinical cases of respiratory failure from non-pulmonary origins; all cases displayed hemodynamic improvement due to reducing the work of breathing through high-velocity therapy prior to receiving definitive therapy for underlying pathologies.

## Introduction

The relationship between work of breathing and hemodynamics is exceedingly complex, and the relationship is dynamic throughout the course and severity of illness. Moreover, actual measurement of work of breathing is difficult in a clinical setting, particularly in a non-mechanically ventilated patient. These challenges have led to clinicians using surrogate clinical signs as indicators of increased work of breathing including respiration rate, use of accessory muscles, and voluntary changes in body position. Clinical signs of high work of breathing (WOB) can be key early indicators of poor prognosis. However, these signs are often poorly recognized when not associated with hypoxemia.

While it is impossible to quantify the prevalence of increased work of breathing due to illness, dyspnea is the chief complaint for roughly 4 million emergency department (ED) visits annually in the United States (1). The etiology of dyspnea is not limited to the pulmonary system and may results through interactions of multiple organ systems and underlying pathophysiology, including the cardiovascular system, hematologic abnormalities, neurological illnesses, metabolic irregularities, and psychogenic causes (2). Metabolic acidosis leads to compensatory increase in tidal volume and respiratory rate (3). Regardless of the etiology in critically ill patients, the necessary cardiac output and oxygen consumption can increase the metabolic cost of breathing by up to 25% (4, 5). One of the potential repercussions of sustaining a high metabolic cost of breathing is that blood may be shunted from the heart, brain, liver, kidneys, and gastrointestinal system ultimately contributing to multi-organ system failure (6).

Efforts to quantify work of breathing non-invasively using readily available clinical data have yielded several clinical tools and scoring systems (7–10). Respiratory rate is one element included in all such scoring systems, and a growing body of literature suggests that evidence of increased breathing work may be one of the most important prognostic indicators for identifying patients at risk for critical illness and poor outcomes (11–14). Increased respiratory rate has been shown to predict cardiac arrest/failure and in-hospital mortality (11, 13, 14).

When a patient is identified as exhibiting signs of increased work of breathing, the most common approach is to provide supplemental oxygen, usually by low-flow nasal cannula. However, this intervention alone may not be sufficient (15). It is imperative to recognize that neither dyspnea nor increased work of breathing are equivalent to hypoxia. While hypoxemic patients benefit from oxygen supplementation, additional oxygen alone has not been shown to improve clinical outcomes in non-hypoxic patients (15–18). Collectively, clinicians have focused on making oxygen delivery supranormal with hyperoxia, fluids, blood transfusion, and vasoactive drugs; however, this has been unsuccessful in improving patient outcomes and may pose potential harms (15, 18–21).

Perhaps, the focus of care for these patients should be broader. Instead of simply providing additional oxygen, supporting improved gas exchange may be more clinically significant. When clinicians increase the efficiency of gas exchange, work of breathing and total oxygen consumption (VO_2_) decrease, making oxygen delivery (DO_2_) more efficient. This concept is well-described in mechanically ventilated critically ill patients (6), but more recent advances may allow support of patients earlier in the course of illness using noninvasive high flow nasal oxygen (HFNO) systems. Broadly, HFNO utilizes flow rates up to 60+ L/min of air with FiO_2_ up to 100%. High-velocity nasal insufflation (HVNI) is a subtype of HFNO that utilizes a small-bore nasal cannula with lower flow rate. The gas has greater kinetic energy leading to a larger flush of the large airways (22) and a different FDA classification (DEN170001). The clinical impact of these mechanistic differences remains unclear.

Recent data suggest that HFNO usage in sepsis and septic shock patients significantly decreased respiratory effort and drive compared to low-flow oxygen users (23). Therefore, early HFNO usage in patients with increased work of breathing may lead to improved patient outcomes. Here, we present three cases of patients who were supported with HVNI prior to definitive therapy for various underlying pathologies. All displayed improvement of vital signs and lactate levels, presumably through reduced work of breathing.

## Case description

### Case 1

A 23-year-old female with diabetes mellitus type-1 presented to the ED with abdominal pain, vomiting, and dyspnea. On initial presentation, the patient was afebrile with the following vital signs: blood pressure (BP) 103/64 mmHg, heart rate (HR) 130 beats/min, and respiratory rate (RR) 36 breaths/min. Despite a normal SpO_2_, this patient had severe increased work of breathing evidenced by tachypnea, use of accessory respiratory muscles, and thoracoabdominal asynchrony. Pertinent lab findings showed hyperglycemia to 1,608 mg/dL, hypokalemia to 2.9 mmol/L, Bicarbonate < 5 mmol/L, anion gap of 29 mmol/L with an initial lactate of 8.6 mmol/L. Initial arterial blood gas (ABG) showed an uncompensated metabolic acidosis or a metabolic acidosis with a subtle respiratory acidosis. Given the patient's tachypnea in the setting of metabolic derangements, the decision was made to administer HVNI at a flow rate of 40 L/min and 21% FiO_2_ to offload the patient's work of breathing. After 10 min, the patient's HR decreased to 107 beats/min and her RR decreased to 25 breaths/min; repeat blood gas was obtained 30 min after HVNI administration, which displayed improvements in acidosis (pH 7.15), bicarbonate of 15 mmol/L, and lactate of 4.5 mmol/L. These improvements occurred prior to the administration of insulin, fluids, or potassium chloride. Supporting the patient's respiratory efforts and reducing work of breathing using HVNI stabilized the clinical situation, which allowed the clinical team to focus on source control.

### Case 2

A 51-year-old female with alcoholic cirrhosis presented to the ED with hematemesis and abdominal distension. The patient was initially hypotensive (80/38 mmHg), tachycardic (127 beats/min), and tachypneic (35 breaths/min). Pertinent labs included a hemoglobin of 8.4 g/dL, bilirubin of 16 mg/dL, bicarbonate of 8 mmol/L, PaCO_2_ of 30 mmHg, lactate of 11 mmol/L, and pH 7.16. The patient was started on HVNI with a flow rate of 40 L/min and 50% FiO_2_. Within 10–15 min, the patient's heart rate decreased to 103 beats/min, RR improved to 23 breaths/min, PaCO_2_ decreased to 18 mmHg, and lactate improved to 8 mmol/L. Improvements all occurred prior to definitive therapy for esophageal and gastric varices (including, but not limited to, blood transfusion, administration of ceftriaxone and octreotide, and esophagogastroduodenoscopy).

### Case 3

A 38-year-old male with hypertension, type 2 diabetes mellitus, heart failure with preserved ejection fraction (HFpEF), sleep-disordered breathing (combined obstructive sleep apnea and obesity hypoventilation syndrome), and severe obesity (BMI > 60) presented to the ED with confusion. Initial vitals included a blood pressure of 105/48 mmHg, HR 86 beats/min, and RR 28 breaths/min. Pertinent labs were noted for WBC 13 × 10^9^/L, sodium 128 mmol/L, bicarbonate 27 mmol/L, creatinine 4 mg/dL, and a lactate of 4.8 mmol/L. ABG showed a pH of 7.35 and PaCO_2_ of 53 mmHg. After initiation of HVNI at a flow rate of 30 L/min and 50% FiO_2_, the patient's heart rate improved to 80 beats/min, RR reduced to 23 breaths/min, and lactate improved to 2.2 mmol/L after 10–15 min, prior to the initiation of definitive therapy.

## Discussion

Herein we report three distinct cases where mitigating work of breathing by HVNI during source control management likely prevented the need for mechanical ventilation and improved clinical status. Signs of increased work of breathing should be more readily considered to be an early warning sign for the decompensation of hospitalized patients (24, 25). Therefore, we believe clinicians should begin to equate clinical signs of increased work of breathing with the need to initiate gentle supportive therapy. If possible, this intervention should occur even prior to obvious severe acidosis.

Changes in the work of breathing are of particular importance in critical illness. At rest, the proportion of cardiac output required to support breathing is negligible. However, work of breathing can require up to 25% of the total cardiac output in critical illness (5). Reducing the work of breathing may alleviate some of the physiologic stress of the patient, preventing fatigue and loss of reserve capacity. High flow nasal oxygen has been shown to flush large airways, functionally reduce anatomic dead space, and reduce work of breathing (25–27). A randomized crossover study of 12 patients under four different conditions showed significant reduction in work of breathing with HFNO at 60 L/min. Esophageal pressure variation, esophageal pressure-time product/min, and work of breathing/min all decreased significantly while dynamic lung compliance increased (28). Additionally, Mauri et al. studied 25 patients with extrapulmonary sepsis or septic shock and found that, when compared with low flow oxygen, treatment with HFNO led to significantly decreased respiratory effort and respiratory drive (23).

In a multicenter study of patients with moderate or severe COPD, treatment with HVNI reduced RR by 28% and significantly reduced PaCO_2_ and accessory muscle usage (29). This suggests that HVNI improves gas exchange even in those with significant ventilatory impairment. These data, in combination with the cases reported here, provide early evidence that HVNI can reduce work of breathing. This support may slow the progression of decompensation in some patients with critical illness until definitive therapies take effect. Collectively, these cases raise the hypothesis that expanded use of HFNO may benefit some patients with non-pulmonary causes of severe illness who exhibit signs of increased work of breathing.

## Conclusions

The cases described illustrate the concept that clinicians should consider respiratory support early in the care of patients with evidence of increased work of breathing, even if the underlying cause is not pulmonary in origin and/or there is no overt hypoxemia or hypercapnia. Early intervention with HFNO is a non-invasive way to improve gas exchange, supporting the correction of metabolic acidosis as well as offloading the percentage of cardiac output that is being utilized for breathing. This concept warrants further study to identify appropriate patient populations and treatment strategies as well as characterize potential clinical and economic impacts.

## Data availability statement

The original contributions presented in the study are included in the article/supplementary material, further inquiries can be directed to the corresponding author.

## Ethics statement

Ethical review and approval was not required for the study on human participants in accordance with the local legislation and institutional requirements. Written informed consent for participation was not required for this study in accordance with the national legislation and the institutional requirements.

## Author contributions

JG and JW conceptualized the manuscript. CS, JG, KW, and JW participated in the development and writing of this manuscript, and all authors agree to be accountable for the content of the work. All authors contributed to the article and approved the submitted version.

## Conflict of interest

JW is VP of Clinical Research for Vapotherm, Inc.—a manufacturer of high flow oxygen systems. KW has been employed within the past 12 months as a scientific consultant. JG has served as a consultant to Vapotherm, Inc. within the last 3 years for the development of educational materials. The remaining author declares that the research was conducted in the absence of any commercial or financial relationships that could be construed as a potential conflict of interest.

## Publisher's note

All claims expressed in this article are solely those of the authors and do not necessarily represent those of their affiliated organizations, or those of the publisher, the editors and the reviewers. Any product that may be evaluated in this article, or claim that may be made by its manufacturer, is not guaranteed or endorsed by the publisher.
